# Sensory cortical response to uncertainty and low salience during recognition of affective cues in musical intervals

**DOI:** 10.1371/journal.pone.0175991

**Published:** 2017-04-19

**Authors:** Fernando Bravo, Ian Cross, Emmanuel Andreas Stamatakis, Martin Rohrmeier

**Affiliations:** 1 Centre for Music and Science, University of Cambridge, Cambridge, United Kingdom; 2 Institut für Kunst- und Musikwissenschaft, TU Dresden, Dresden, Germany; 3 Division of Anaesthesia, University of Cambridge, Cambridge, United Kingdom; Max Planck Institute for Human Cognitive and Brain Sciences, GERMANY

## Abstract

Previous neuroimaging studies have shown an increased sensory cortical response (i.e., heightened weight on sensory evidence) under higher levels of predictive uncertainty. The signal enhancement theory proposes that attention improves the quality of the stimulus representation, and therefore reduces uncertainty by increasing the gain of the sensory signal. The present study employed functional magnetic resonance imaging (fMRI) to investigate the neural correlates for ambiguous valence inferences signaled by auditory information within an emotion recognition paradigm. Participants categorized sound stimuli of three distinct levels of consonance/dissonance controlled by interval content. Separate behavioural and neuroscientific experiments were conducted. Behavioural results revealed that, compared with the consonance condition (perfect fourths, fifths and octaves) and the strong dissonance condition (minor/major seconds and tritones), the intermediate dissonance condition (minor thirds) was the most ambiguous, least salient and more cognitively demanding category (slowest reaction times). The neuroscientific findings were consistent with a heightened weight on sensory evidence whilst participants were evaluating intermediate dissonances, which was reflected in an increased neural response of the right Heschl’s gyrus. The results support previous studies that have observed enhanced precision of sensory evidence whilst participants attempted to represent and respond to higher degrees of uncertainty, and converge with evidence showing preferential processing of complex spectral information in the right primary auditory cortex. These findings are discussed with respect to music-theoretical concepts and recent Bayesian models of perception, which have proposed that attention may heighten the weight of information coming from sensory channels to stimulate learning about unknown predictive relationships.

## Introduction

We face various forms of uncertainty in our everyday interaction with our environment. Inferences made under uncertainty can occur when either prior information is incomplete or when the outcomes are unclear [1]. According to one influential framework uncertainty may enhance attention to the environment, facilitating processes of associative learning [2,3]. One way in which attentional mechanisms can reduce uncertainty is through the amplification of the signal stimulus [4–6]; in other words, uncertainty may increase attention demands, which in turn may modulate a sensory cortical response. The present study was aimed at investigating attentional enhancement of sensory signals for ambiguous music-evoked emotions [7,8]. We used sound stimuli of distinct levels of consonance/dissonance within a sound-based emotion recognition paradigm, and functional magnetic resonance imaging (fMRI) to investigate the neural correlates for ambiguous affective cues signalled by musical information.

Over the past decades, Bayesian statistical theory has been applied to cognitive processes in perception, and has provided a valuable quantitative framework for its investigation [2,9–14]. The Bayesian perspective proposes that optimal learning and inference crucially rely on representing and processing the different forms of uncertainty associated with a behavioural context. A context is defined as a set of correlational relationships (i.e. statistical regularities) linking objects and events, which allow making inferences about the environment based on prior observations that serve as predictive cues [14]. Predictive coding models propose that activity in neurons within higher stages (higher level areas) are actively attempting to “explain” incoming information represented in lower areas (primary sensory cortices) via feedback projections [15,16]. Visual perception studies have consistently shown that activity in early visual areas is reduced whenever individual features of an image are perceived as coherent patterns or shapes compared to randomly arranged visual elements [17–19]. In the study by Murray and collaborators [19] significant activity increases were observed in the lateral occipital complex (a higher visual area critical for object shape perception), and concurrent reductions of activity were found in the primary visual cortex in response to visual elements that could be grouped into coherent shapes (i.e. lines that form 2D shapes) compared to randomly arranged visual elements (i.e. lines created by breaking the 2D shapes). Evidence suggests that these effects could be relevant for inferential processes, contributing to the disambiguation of sensory inputs [20]. Moreover, empirical evidence has shown that stimuli with greater predictive uncertainty (e.g. visual elements that cannot be perceived as coherent shapes) may suppress the use of prior cues for making inferences (i.e. top-down expectation driven information from higher level areas) compared with direct sensory information, and further stimulate learning about the unknown predictive relationships through increasing the gain of sensory-induced signals [11].

Recent Bayesian models of perception have proposed that attention may heighten the weight of perceptual sensory evidence, reversing the effect of prediction in silencing sensory signals [15,16,21,22]. This framework converges with the signal enhancement theory [4,22] and proposes that attention could be thought as “highlighting” operation conducted on a certain region of the space, which enhances the precision of information coming from this region. A high sensory precision will therefore increase the influence of ascending prediction errors by turning up the “volume” of sensory channels in which more confidence is placed [22,23]. Several neuroimaging studies have provided evidence for attentional enhancement of neural activity in the human visual cortex employing visual paradigms such as the Posner task [24–32]. Fewer empirical studies have been conducted to assess enhanced precision of sensory evidence in the auditory domain (for previous brain imaging studies observing attentional modulation of auditory cortex see: [33–37]). To our knowledge, no studies have so far investigated early sensory cortical responses to uncertainty in the context of music-evoked emotions.

In the present study, information conveyed through sound acted as the non-verbal cue to be interpreted. Subjects had to make temporary valence inferences [38] based on auditory signals that only differed in terms of consonance/dissonance level, which was controlled by interval content manipulation. The task employed a purposely-made metaphor, which informed the participants that a radio-telescope had captured a series of radio signals from outer space. Participants were asked to listen to these signals, and to think and decide if they were produced by good-friendly or bad-aggressive aliens (for details see Methods). The task therefore required to *predict the affective value of a message conveyed via musical intervals*. In the present study, participants’ judgements were considered as inferences of transitory states or temporary inferences [38,39], which have been found to rely on theory of mind function [38,40–44]. It has been argued that expectations about the precision of sensory inputs may play a central role beyond the dynamics of perception, affecting also higher cognitive functions such as social judgments and theory of mind processes [23]. In the context of our task, we define uncertainty (and ambiguity) as the difficulty to evaluate the affective valence of the sound stimuli. We specifically employ the notion of “predictive” uncertainty to refer to participants’ ascription of temporary inferences (i.e. prediction of the affective value of a message) based on nonverbal sensory inputs (for other studies requiring temporary mental state predictions based on nonverbal information see: [40,41,44–46]).

This study concentrates on the effects elicited by consonance/dissonance, which was controlled by manipulating the interval content of algorithmically generated sounds. Various psychoacoustic models have been suggested to elucidate why musical intervals comprising simple frequency ratios, such as the octave (2:1) or the perfect fifth (3:2), are experienced as more consonant than intervals involving complex ratios such as the major second (9:8), the minor second (16:15) or the tritone (45:32) [47–51]. One influential theory was coined by Helmholtz [47], who proposed that sensory consonance/dissonance was associated with the absence/presence of interactions (sensation of “beats” or “roughness”) between the harmonic spectra of two pitches [49]. At a physiological level, it has been argued that beating emerges when two or more simultaneous components of a complex sound are kept apart from one another in frequency by less than the width of an auditory filter or ‘critical band-width’ (10–20% of center frequency) [52], becoming unresolved by the auditory system [53]. However, empirical evidence has also shown that the perception of consonance/dissonance can be elicited not only by the properties of a single signal, such as roughness/beating, but also when tones are presented dichotically [54–57]. In dichotic listening tasks different pitches are presented separately to each ear, which avoids cochlear interactions (e.g. for dichotic dissonance: a consonant signal is presented to each ear but both stereo signals differ by a minor second) [54,58–65]. Fritz and collaborators [56], have shown that dichotic dissonance stimulation also elicits negative valence ratings, which indicates that cochlear interactions may not be critical for the perception of dissonance. It is important to note, however, that when notes are presented dichotically, the allocation of attention in the auditory space can be modulated by training [66] and, consequently, participants’ valence judgments during dichotic paradigms could also be explained by attentional focus on one ear. To overcome this potential problem, in the present work we employed *sequential* intervals presented diotically (each tone was audible by both ears simultaneously), which do not produce roughness or beats due to their non-simultaneity; yet sequential intervals are also known to be judged along the dimension of consonance/dissonance according to their frequency ratios [56,57,67,68]. Task conditions for the experiment comprised three sound categories: a consonant condition (interval content–henceforth i.c.–: perfect fourths, perfect fifths and octaves), an intermediate dissonant condition (i.c.: minor thirds) and a strong dissonant condition (i.c.: minor seconds, major seconds and tritones). All three conditions were based on music theoretically and psychologically established concepts of consonance and dissonance [49,69,70]. Following on from previous evidence [68,71,72] we hypothesised that participants’ valence inferences would be influenced by the level of consonance/dissonance, with consonant sounds leading to positive interpretations of the auditory signals (i.e. good-friendly), whilst increasing levels of dissonance would guide participants towards ambiguous (intermediate dissonant condition) and negatively valenced inferences.

Importantly, the focus of the present study was centred on the intermediate dissonant condition, which was constructed based on the sonority elicited by the diminished seventh chord, a four note harmonic set consisting of three minor thirds above the root [73,74]. The diminished seventh chord has been frequently used in tonal music to connote affective states of suspense and ambivalence, specially in the Baroque era and in the early years to the 19^th^ century [7,8]. The intermediate dissonant condition was thus created employing sequentially triggered minor thirds. Because of its intermediate position with respect not only to consonance/dissonance [49,75] but also to tonalness level [76], we predicted that this category would be the most ambiguous condition, and that its implied uncertainty in terms of valence attribution would be reflected in higher cognitive processing demands, and further lead to heightened weighting of sensory evidence in an attempt to improve the quality of the stimulus representation. The two experiments reported in this article resulted in rich data sets; we here constrain our scope to those aspects that directly relate to the described predictions.

## Material and methods

### Subjects

#### Experiment 1a (behavioural study-United Kingdom)

Forty-five individuals participated in the laboratory experiment conducted in Cambridge (UK) (22 women, 23 men; mean age = 18.4, SD = 1.9). Subjects reported no long-term hearing impairment. None of the participants was a professional musician. Eight participants reported having received informal musical training for less than three years; the other 37 participants did not receive any musical training. All subjects gave informed consent. The study received ethical approval from the Music Faculty Research Ethics Committee (University of Cambridge).

#### Experiment 1b (behavioural study-Argentina)

Thirty individuals participated in the laboratory experiment conducted in Buenos Aires (Argentina) (15 women, 15 men; mean age = 28.9, SD = 1.9). Subjects reported no long-term hearing impairment. None of the participants was a professional musician. Five participants reported having received informal musical training for less than three years; the other 15 participants did not receive any musical training. All subjects gave informed consent. The study received ethical approval from the Music Faculty Research Ethics Committee (Universidad Católica Argentina).

#### Experiment 2 (fMRI study)

Data were obtained from twelve subjects (7 females, 5 males; mean age = 29, SD = 5.16). All participants were right-handed volunteers with no self-reported neurological or psychiatric conditions from Fundación Científca del Sur Imaging Centre (FCS) community (radiology residents, radiographers and administrative personnel). None of the participants was a professional musician. Two participants reported having received informal musical training for less than three years; the other ten participants did not receive any musical training. All subjects gave informed consent. The study received ethical approval from FCS.

### Stimulus material and design

Auditory stimuli construction: Research examining inferences made under uncertainty use diverse approaches to define and control uncertainty [77]. Our paradigm entailed a fine-grain emotion recognition task in which participants were presented with sequences of sounds triggered in rapid succession (7 notes per second; note duration = 128 milliseconds). We controlled uncertainty in participants’ valence inferences by manipulating the level of consonance/dissonance of the experimental stimuli.

We employed sequential intervals, which do not produce roughness or beats due to their non-simultaneity, yet they are also known to be judged along the dimension of consonance/dissonance according to their frequency ratios [67,68]. Numerous psychoacoustic models have been proposed to explain the consonance/dissonance percept [47–51,55,57]. According to these models, the most consonant intervals would be the ones that could be expressed with simple frequency ratios, which has been supported by psychological studies [75,78,79]. Intervals such as the unison (1:1), the octave (2:1), perfect fifth (3:2), and perfect fourth (4:3) are regarded as the most consonant. Intermediate in consonance are the major third (5:4), minor third (6:5), major sixth (5:3), and minor sixth (8:5). The most acoustically dissonant intervals (composed of frequencies the ratio between which is not simple) are the major second (9:8), minor second (16:15), major seventh (15:8), minor seventh (16:9), and the tritone (45:32). The sounds for the experiment were created using pure tone sequences, and systematically manipulated through algorithms, by means of which the three distinct levels of consonance/dissonance were generated. The consonance condition employed a highly consonant interval content (perfect fourths, perfect fifths, octaves), the intermediate dissonance condition was built based on the diminished triad, which was assumed to elicit moderate dissonance effects (minor thirds); finally, the strong dissonance condition was constructed with a highly dissonant interval content (minor seconds, major seconds, tritones). Within sound conditions, musical intervals were triggered in sequential order (e.g. strong dissonance condition: 1^st^–minor second, 2^nd^–major second from last triggered note, 3^rd^–tritone from last triggered note / mod.12). The present study was specifically centred on the effects elicited by the intermediate dissonant condition. Table 1 shows the three pitch-class sets that were employed in this experiment (which correspond to the three sound conditions described above) together with their respective tonalness values. The notion “tonalness” has been defined as “the degree to which a sonority evokes the sensation of a single pitched tone” [80] in the sense that sonorities with high tonalness evoke a clear perception of a tonal center [81]. Temperley [76] has suggested a way to calculate tonalness level, following a Bayesian ‘structure–and-surface’ approach, as the overall probability of a pitch-class set occurring in a tonal piece. Previous empirical evidence indicates that the tonalness level of a sonority could represent a quantifiable predictor of emotional valence associations [72].

**Table 1:** Tonalness values for the three sound conditions.

Interval Set/ Prime Form	Tonalness
Consonance [5,7,12]/(0,2,7)	0.00231
Intermediate dissonance [3]/(0,3,6)	0.00032
Strong dissonance [1,2,6]/(0,1,2,6)	0.00016

Tonalness values for the three sound conditions employed in the experiment calculated using the Kostka-Payne key-profiles (Numbers in brackets indicate interval set, numbers in parenthesis denote the correspondent prime form).

The distinctive impact of each sound category on valence ratings had been preliminary validated in a pilot study with 26 naive normal subjects (age range: 26–30), tested during the 2013 Cambridge’s Festival of Ideas. Results indicated that participants did rate the three sound conditions differently in the valence dimension, Wilks’ Lambda F (3, 23) = 8.266, p = 0.001. A significant difference was observed between strong dissonant sounds (mean: 7.19 -negative valence-, SD: 3.28) and consonant sounds (mean: 3.85 -positive valence-, SD: 2.90), (F (1, 25) = 13.632, p = 0.001).

**Fig 1:**
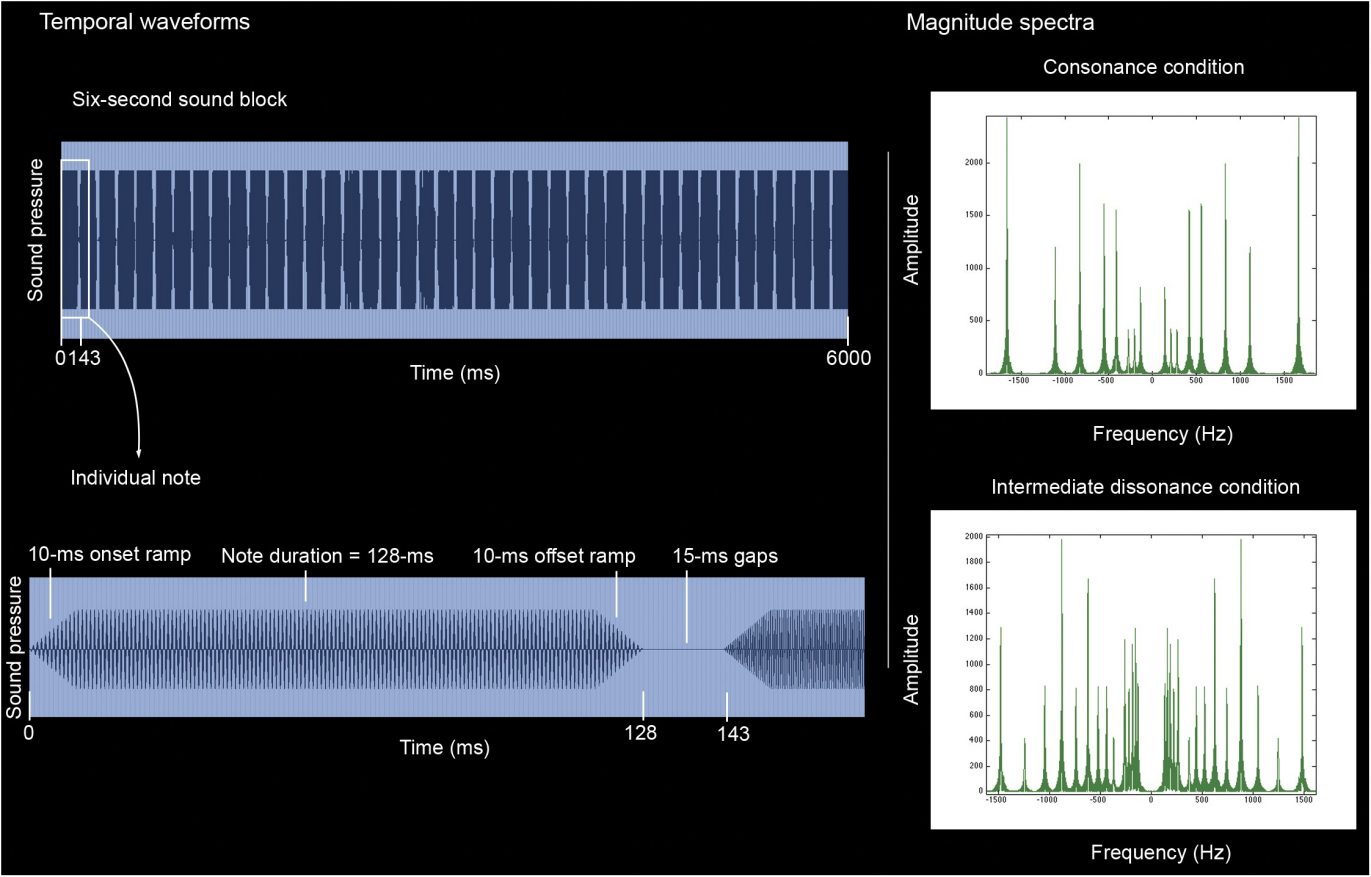
(Left) Time waveforms (High-resolution images; gridlines represent milliseconds). Each six-second sound block (Upper blue representation) consisted of 41 musical interval presentations (42 individual notes) for a particular condition. Each note (Lower blue representation) had a total duration of 128 milliseconds (ms), including 10-ms raised-cosine onset and offset ramps. Notes were separated by 15-ms gaps, producing an overall presentation rate of 7 notes per second. (Right) Spectral representation for a six-second sound block [Matlab, plotted Fourier transform expression fft(x)] belonging to the consonance condition and to the intermediate dissonance condition. Abbreviations: Hz: Hertz.

Description of the sounds (Fig 1): Within a sound block, each note had a total duration of 128 milliseconds (ms), including 10-ms raised-cosine onset and offset ramps, and was triggered with a fixed velocity (i.e. constant loudness). Notes were separated by 15-ms gaps, producing an overall presentation rate of 7 notes per second (42 notes = 41 musical intervals per six-second sound block). Although very short tones employed, within the register utilized (70–1600 Hertz), evidence indicates that individuals can discriminate differences in their frequencies [82,83].

Design: A repeated measures design was employed. The manipulated (independent) variable was the level of consonance/dissonance (consonance, intermediate dissonance and strong dissonance). The outcome (dependent) variable was participants’ ratings in terms of valence inferences (positive or negative). We additionally evaluated the salience for the valence judgments by examining how the ratings for each sound condition deviated from a neutral valence value (11-point scales were employed, neutral valence was defined as test value = 6). Repeated measures ANOVAs were used to examine whether there were differences between the valence ratings, and between the reaction times, for the three sound conditions. When appropriate, post hoc contrasts (corrected for multiple comparisons) were conducted to determine the nature of these effects by comparing which pairs had significantly different means.

**Fig 2:**
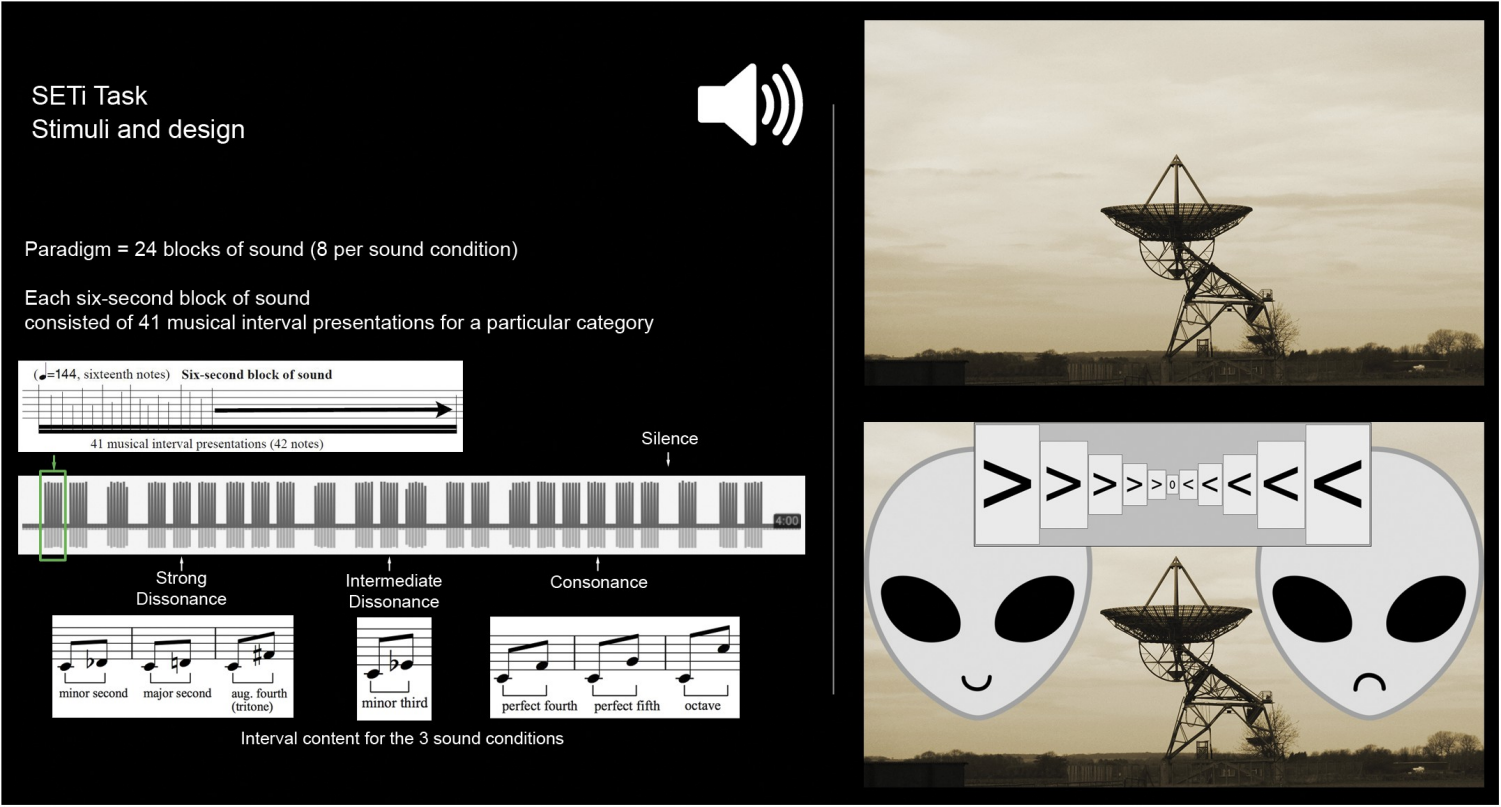
(Left) Sound stimuli and paradigm design. (Right) Laboratory version of the experiment: subjects viewed the above image of a radio-telescope and were given the following instruction: “A radio-telescope located in Cambridge captured a series of radio signals from outer space. You will listen to these sounds and your task is to think and decide if they were produced by good-friendly or bad-aggressive aliens”. Participants had to select their answer for each sound block using an 11-point scale (mouse click), which appeared onscreen immediately after a sound block was played (the side of ‘good’ and ‘bad’ alien was semi-randomized).

The complete experiment involved 24 blocks of sound (Fig 2). Each six-second block of sound consisted of 41 musical interval presentations (42 individual notes) for a particular condition (Fig 1). The paradigm comprised 8 blocks of sound per condition, totalling 328 musical interval presentations per sound category. This was done in order to reliably estimate the haemodynamic response function (HRF) and to show detectable differences between conditions in the neuroscientific setting. Each six-second block of sound started with a distinct, randomly assigned, initial pitch (i.e. each sound block was unique), but which belonged to the intervallic-content set determined by each sound condition. To examine whether valence was balanced across sound categories, the stimuli were piloted before testing. No significant differences were found in the valence judgments for sound blocks belonging to the same sound condition. Cronbach’s alpha was further computed (equivalent forms reliability) to assess whether each subset of eight sounds ratings, which were averaged to create the composite ratings, formed a reliable measure. The alpha values for the consonant sounds (0.761), for the intermediate dissonant sounds (0.813), and for the strong dissonant sounds (0.780) indicated that the ratings for the sounds corresponding to the same consonance/dissonance level had reasonable internal consistency, supporting the core theoretical strategy underlying this study’s experimental design, which assumed that the ratings that were combined to conform a specific composite value belonged to the same consonance/dissonance level (and corresponded to sounds created with the same interval content). A silent condition was added with 8 presentations of six seconds each (blocks of rest), acting as a baseline. Sound blocks were separated by two seconds of silence (inter-trial interval), unless there was a silent condition in between two sound blocks, in which case no additional separation time was included. No repetitions of silence were allowed, and there were never more than two consecutive sound blocks belonging to the same level of consonance/dissonance. Four different pseudo-randomized orderings of the sound blocks were utilised, in which sound blocks were carefully distributed to avoid contrasting trials that are far apart in time in the fMRI analysis.

### Procedure

The same task, using exactly the same stimuli, was carried out in both experimental settings (i.e. laboratory and fMRI). Subjects were asked to make valence inferences based on non-verbal auditory cues. The task employed a purposely-made metaphor, which informed the participants that a radio-telescope had captured a series of radio signals from outer space. Participants were required to listen to these radio signals (24 blocks of sound), and to “decide if they were produced by good-friendly or bad-aggressive aliens”. In the present study, participants’ judgements were considered as inferences of transitory states or temporary inferences [39], which have been found to rely on theory of mind function [38,40–44]. The paradigm, therefore, required participants to categorize stimuli of different consonance/dissonance level in terms of positive/negative valence.

#### Experiment 1a (laboratory-United Kingdom)

The experiment was run in the Centre for Music and Science (CMS) at the University of Cambridge. All subjects performed the task using the CMS workstations and listened to the stimuli with Behringer HPM1000 Headphones. Sound pressure levels were measured with a Galaxy Audio CM130 Meter, the output volume was set to be identical in all workstations (average sound level = 70dB). Participants had to select their answer using a multiple-categories rating format; 11-point Likert scales were employed for measuring the dependent variable to obtain higher resolution/more fine-grained scores to perform statistical analyses with (compared to the 5-point scales used in the post-scan questionnaire). The side of ‘good-friendly’ or ‘bad-aggressive’ alien image was semi-randomized (Fig 2). The task was presented through a stand-alone interactive application (programmed by FB in MaxMSP-Cycling’74), which enabled reaction time recordings captured at the millisecond level. Reaction times were measured from the onset of each sound block to the time when the participant made the valence rating. This was captured through MaxMSP’s “mousestate” object, which allowed registering mouse clicks on a mask overlayed onto the rating scale image. Before the testing session, subjects underwent a training session in which they were familiarised with the task and trained on the procedure with nine trials (three per sound condition) with sample stimuli constructed based on the testing materials.

#### Experiment 1b (laboratory-Argentina)

The experiment was run at Universidad Católica Argentina in Buenos Aires (Argentina), in similar conditions as in Cambridge (UK) (i.e. acoustically treated environment with sound absorbing walls). The task was also presented using the software application MaxMSP (Cycling’74).

#### Experiment 2 (fMRI study)

Participants were asked to arrive to the Imaging Centre 45 minutes before the fMRI scanning session, in order to undertake the training session of 10 minutes in a separate room (contiguous to the scanner room). Subjects were familiarised with the task and trained on the procedure with nine trials (three per sound condition) with sample stimuli constructed based on the testing materials. Participants were instructed to think and decide on a response to the task question as soon as they heard the onset of each of the sound blocks, which were separated by blocks of silence.

In the fMRI setup, the visual stimuli (invariant still image of a radio-telescope) was projected onto a screen and presented to the subject via a 45° angled mirror positioned above the participant’s head. Subjects were given the same instruction as in the behavioral study, but they were asked to produce a covert response (i.e. “…You will listen to these sounds and your task is to think and decide if they were produced by good-friendly or bad-aggressive aliens…”). An MRI-compatible response collection system was not available at the Imaging Centre in Argentina and, therefore, subjects’ RTs could not be collected in the neuroscientific setting (separate behavioral experiments were conducted from a population similar to the fMRI participants in the UK and in Argentina for this purpose). The auditory stimuli were delivered via Etymotic ER30 tube-phones (Etymotic Research, Illinois, USA). Following the scanning session each subject underwent the behavioral version of the experiment (same ordering of sound stimuli as inside the MRI scanner). Subject-specific behavioral data was collected through a paper-based questionnaire (5-point Likert scales), which was subsequently related to the functional imaging data.

### fMRI data acquisition

A General Electric Signa system operating at 3 Tesla was utilized. Prior to the functional magnetic resonance measurements, high resolution (1 x 1 x 1 mm) T1-weighted anatomical images were acquired from each participant using three-dimensional fast spoiled gradient- echo (3D-FSPGR) sequence. Continuous Echo Planar Imaging (EPI) with blood oxygenation level-dependent (BOLD) contrast was used with a TE of 40ms and a TR of 3000ms. The matrix acquired was 64 x 64 voxels (in plane resolution of 3 mm x 3 mm). Slice thickness was 4 mm with an interslice gap of 0.7 mm (35 slices, whole brain coverage). Functional images were acquired over one run of 4 minutes. The sound files used for the task were digitally recorded onto compact disks and delivered to participants at a loudness level equal for all subjects.

### fMRI data analysis

Data were processed using Statistical Parametric Mapping (SPM), version 8 (Wellcome Department of Imaging Neuroscience, London, UK—http://www.fil.ion.ucl.ac.uk/spm). Following correction for the temporal difference in acquisition between slices, EPI volumes were realigned and resliced to correct within subject movement. A mean EPI volume was obtained during realignment and the structural MRI was coregistered with that mean volume. The coregistered structural scan was normalized to the Montreal Neurological Institute (MNI) T1 template [84]. The same deformation parameters obtained from the structural image, were applied to the realigned EPI volumes, which were resampled into MNI-space with isotropic voxels of 3 cubic millimeters. The normalized images were smoothed using a 3D Gaussian kernel and a filter size of 6 mm FWHM. A temporal highpass filter with a cutoff frequency of 192 Hz was applied with the purpose of removing scanner attributable low frequency drifts in the fMRI time series.

An event-related design was modeled by using a canonical hemodynamic response function (HRF). The design matrix included the following four regressors: consonant sounds, strong dissonant sounds, intermediate dissonant sounds and rest (baseline). Parameter estimate images were generated. Nine contrast images per individual were calculated: cons > rest, intermediate diss > rest, strong diss > rest, cons > intermediate diss, cons > strong diss, intermediate diss > cons, intermediate diss > strong diss, intermediate diss > cons and strong diss > intermediate diss.

After performing a one-way analysis of variance (ANOVA), which showed a significant overall effect of the experimental manipulation (data in S1 Table), second level group analyses were carried out using one-sample t-tests, to assess the specific ways in which the means for each condition differed. The significant map for the group random effects analysis was thresholded at voxel level *p* < 0.001 uncorrected, with a cluster level threshold of *p* < 0.05 corrected for a selected regions of interest (ROIs) using family wise error (FWE). Following meta-analytic reviews (statistical summaries of empirical findings across studies) and previous neuroscientific studies that have investigated the auditory processing of complex spectral information, in the present study we examined signal changes on the bilateral primary auditory cortex, which has consistently shown differential sensitivity to consonant and dissonant pitch relationships [85–89]. Small volume correction was also applied to signal changes observed in core regions of the salience and ventral attention networks including the right temporo-parietal junction, ventral frontal cortex and bilateral anterior insula [26,90]. All ROIs were defined using anatomical masks of the described areas with WFU PickAtlas Toolbox [91].

Psycho-physiological interactions (PPI) analysis: Following the approach developed by Friston et al. [92] functional connectivity was measured in terms of psycho-physiological interactions (PPI). A seed region of interest in the right Heschl’s gyrus was selected on the basis of significantly activated clusters from the subtractive analysis comparing intermediate dissonance against the consonance condition. The group cluster peak (i.e. intermediate dissonance > consonance: MNI coordinates 48–10 7) was used as point of reference to identify individual subject activation peaks that complied with the following two rules: a) were within a 24 mm radius, and b) were within the boundaries of the corresponding brain area created using the WFU pickatlas toolbox [91]. After the identification of the relevant statistical peaks for each subject, a sphere was defined around these peaks with a 6 mm radius, which were used as the seed regions of interest for the PPI analysis. This type of analysis is used to detect target regions for which the covariation of activity between seed and target regions is significantly different between the experimental condition of interest: intermediate dissonance > consonance. For each seed ROI, the contrast images from all subjects were used in voxel-wise one-sample *t*-tests at the second level (at threshold level *p* < 0.001 voxel uncorrected, *p* < 0.05 cluster FWE-corrected).

## Results

### Behavioural experiments (laboratory and post-scan questionnaire)

Forty-five subjects performed the sound-based task in a controlled laboratory setting (United Kingdom). A repeated measures ANOVA was conducted to assess whether there were differences between the average ratings for the three sound conditions. Mauchly’s test of sphericity was not significant (*p* = 0.405; sphericity assumption met). Results indicated that participants did rate the three sound conditions differently, *F*_2, 88_ = 13.103, *p* < 0.001. Post hoc contrasts, with Bonferroni correction, showed that the valence rating for consonant sounds was on average significantly more positive than the valence rating for strong dissonant (*p* = 0.004, *d* = 3.311) and intermediate dissonant sounds (*p* < 0.001, *d* = 2.178). There was no significant difference between the valence rating for strong and intermediate dissonant sounds (*p* = 0.368). Examination of the mean ratings for the three sound categories (listed in Table 2) suggested that participants rated the sounds that consist of more consonant (dissonant) intervals as more positive (negative) in terms of valence, with intermediate dissonances evaluated in-between the two contrasting conditions. Polynomial contrast on the mean ratings for the three sound categories (listed in Table 2) indicated a significant linear trend (*F*_1, 47_ = 13.517, *p* < 0.01), confirming that participants gave more extreme valence ratings to stimuli with more extreme consonant (or dissonant) interval content, whilst intermediate dissonances were evaluated as moderate in valence.

**Table 2:** Valence and reaction time means for the three sound conditions (Laboratory Experiments conducted in the UK and in Argentina).

	UK				Argentina			
Condition	Valence	SD	RT mean	SD	Valence	SD	RT mean	SD
*Intermediate Dissonance*	5.67	3.09	6791.9	6302.5	6.30	2.20	6993.3	4019.0
*Strong Dissonance*	6.80	3.29	4601.4	3189.1	7.10	2.99	4908.4	3411.3
*Consonance*	3.49	2.38	4333.3	4219.4	3.57	2.38	4220.0	3227.5

Valence means with standard deviations (SD) (higher values denote more negative valence; 11-point scales). Reaction time (RT) means and standard deviations (in milliseconds) corresponding to the intermediate dissonance, strong dissonance and consonance conditions.

One-sample *t* tests were conducted to examine how the valence ratings for each sound condition deviated from a neutral valence value (since we employed 11-point scales, neutral valence was defined as test value = 6). The valence rating for the consonance condition was significantly more positive (*t*_44_ = 7.078, *p* < 0.001, 95% CI [-3.23, -1.80]) compared to the test value. The valence ratings for the intermediate dissonance condition (*t*_44_ = 0.724, *p* = 0.473, 95% CI [-1.26, 0.59]) and the strong dissonance condition (*t*_44_ = 1.63, *p* = 0.110, 95% CI [-0.19, 1.79]) were not significantly different from the test value.

A repeated measures ANOVA, was conducted to assess whether there were differences between the reaction times (measured in milliseconds) for the three sound conditions. Mauchly’s test of sphericity was not significant (*p* = 0.066; sphericity assumption met). Results yielded significant differences (*F*_2, 88_ = 4.80, *p* = 0.01). Pairwise comparisons with Bonferroni correction indicated that there was a significant difference between the reaction times for intermediate dissonant sounds and consonant sounds (*p* = 0.016, *d* = 2458). No significant differences in reaction times were found when comparing the strong dissonance condition with the intermediate (*p* = 0.103) or consonance conditions (*p* = 1.000). The means and standard deviations for the reactions times are presented in Table 2.

An additional, separate, behavioral experiment was conducted with 30 subjects, from a population similar to the fMRI participants in Buenos Aires (Argentina) with equally controlled laboratory settings as in the UK experiment. A repeated measures ANOVA was conducted to assess whether there were differences between the average ratings for the three sound conditions. Mauchly’s test of sphericity was not significant (*p* = 0.763; sphericity assumption met). Results indicated that participants did rate the three sound conditions differently, *F*_2, 58_ = 14.856, *p* < 0.001. Post hoc contrasts, with Bonferroni correction, showed that the valence rating for consonant sounds was significantly more positive than the valence rating for strong dissonant (*p* = 0.002, *d* = 3.533) and intermediate dissonant sounds (*p* < 0.001, *d* = 2.733). There was no significant difference between the valence rating for strong and intermediate dissonant sounds (*p* = 0.646). Polynomial contrast on the mean ratings for the three sound categories also indicated a significant linear trend (*F*_1, 29_ = 24.942, *p* < 0.001), showing that participants gave more extreme valence ratings to stimuli with more extreme consonant (or dissonant) interval content, whilst intermediate dissonances were evaluated as moderate in valence. The mean ratings for the three sound categories are listed in Table 2.

One-sample *t* tests were conducted to examine how the valence ratings for each sound condition deviated from a neutral valence value (test value = 6). The valence rating for the consonance condition was significantly more positive (*t*_29_ = 5.581, *p* < 0.001, 95% CI [-3.33, -1.54]) compared to the test value. The valence ratings for the intermediate dissonance condition (*t*_29_ = 0.747, *p* = 0.461, 95% CI [-0.52, 1.12]) and the strong dissonance condition (*t*_29_ = 2.017, *p* = 0.053, 95% CI [-0.02, 2.22]) were not significantly different from the test value.

A repeated measures ANOVA, was conducted to assess whether there were differences between the reaction times (measured in milliseconds) for the three sound conditions. Mauchly’s test of sphericity was not significant (*p* = 0.193; sphericity assumption met). Results yielded significant differences (*F*_2, 58_ = 5.09, *p* < 0.001). Pairwise comparisons with Bonferroni correction indicated that there was a significant difference between the reaction times for intermediate dissonant sounds and consonant sounds (*p* = 0.028, *d* = 2773). No significant differences in reaction times were found when comparing the strong dissonance condition with the intermediate (*p* = 0.113) or consonance conditions (*p* = 1.000). The means and standard deviations for the reactions times are presented in Table 2.

These results obtained in Buenos Aires (Argentina) converge and support the pattern evidenced in the behavioural experiment conducted in Cambridge (UK), and therefore suggest that the valence percept of musical intervals is comparable in both testing populations.

Following the scanning session subject-specific behavioral data were collected for the twelve participants who took part of the fMRI study. Convergent with the laboratory experiment, results indicated that participants rated the conditions differently (Wilks’ Lambda *F*_2,10_ = 6.91, *P* = 0.013). Post hoc tests showed that the valence rating for consonant (dissonant) sounds was on average significantly more positive (negative) than the valence rating for strong dissonant (*P* < 0.05, *d* = 0.427), supporting the findings reported for the laboratory experiment. Polynomial contrasts revealed the same significant linear trend for valence ratings (*F*_1,11_ = 12.141, *P* < 0.01), consonance < intermediate dissonance < strong dissonance, although the intermediate dissonant condition did not differ significantly from either of the other two conditions. The means, standard deviations and 95% confidence intervals are listed in Table 3.

**Table 3:** Valence means and 95% confidence intervals for the three sound conditions (post-scan questionnaire).

Condition	Valence	SD	95% CI (adjusted)
*Intermediate Dissonance*	2.94	0.69	[2.629, 3.261]
*Strong Dissonance*	3.09	0.65	[2.847, 3.334]
*Consonance*	2.66	0.45	[2.499, 2.828]

Valence means, standard deviations and 95% confidence intervals for each sound condition (higher values denote more negative valence; 5-point scales). Confidence intervals indicate adjusted values for repeated measures following the method proposed by Loftus and Masson [93].

### Neuroscientific experiment

When contrasting each sound condition against the silent baseline condition, we found similar patterns of brain response involving primary and secondary auditory cortices bilaterally. Table 4A present the findings for these respective contrasts (cluster-level threshold of *p* < 0.05, FWE-corrected for the whole brain volume). Overall, these results converge with previous evidence from studies using sound sequences in listening paradigms [94,95], showing bilateral engagement of primary and secondary auditory cortices when contrasted to silent baselines.

**Table 4:** fMRI results.

Region	Peak MNI	Voxels	Max t-value (z)	Mean t (std.)	*p*-value (FWE)
**A) Subtractive analysis**					
*Consonance > Baseline*					
Temporal Sup R	45–16 4	268	9.35 (4.82)	5.41 (0.97)	< 0.001
Heschl R	48–19 10	44	6.49 (4.08)	6.02 (1.35)	0.003
Temporal Sup L	-45 -22-4	228	4.47 (3.31)	5.24 (0.99)	< 0.001
Heschl L	-36–28 7	21	4.79 (3.45)	4.99 (0.82)	0.020
*Interm*. *Diss*. *> Baseline*					
Temporal Sup R	45–16 4	254	7.91 (4.49)	4.94 (0.72)	< 0.001
Heschl R	48–19 10	48	6.06 (3.94)	5.65 (0.98)	0.003
Temporal Sup L	-57–22 7	194	8.55 (4.64)	5.12 (0.93)	< 0.001
Heschl L	-42–22 7	25	5.70 (3.81)	4.96 (0.72)	0.006
*Strong Diss*. *> Baseline*					
Temporal Sup R	51–7–2	276	8.98 (4.74)	5.11 (0.90)	< 0.001
Heschl R	48–19 10	44	6.15 (3.97)	5.69 (0.99)	0.004
Temporal Sup L	-57–22 7	171	11.02 (5.14)	5.34 (1.11)	< 0.001
Heschl L	-45–28 10	31	4.72 (3.42)	5.23 (1.41)	0.024
*Consonance > Intermediate dissonance*			No suprathreshold signal changes		
*Consonance > Strong dissonance*			No suprathreshold signal changes		
*Intermediate dissonance > Strong dissonance*			No suprathreshold signal changes		
*Interm*. *Diss*. *> Consonance*					
Heschl R	48–10 7	13	4.22 (3.19)	3.46 (0.18)	0.033
*Strong Diss*. *> Consonance*					
Angular R	36–58 43	21	7.35 (4.34)	5.16 (0.89)	0.006
Parietal inferior R	36–55 40	5	5.31 (3.66)	4.59 (0.45)	0.041
*Strong Diss*. *> Interm*. *Diss*.					
Insula L	-27 23–8	38	5.77 (3.84)	3.60 (0.53)	0.039
Insula R	48 14–8	6	5.22 (3.63)	4.56 (0.49)	0.049
Anterior Cingulate R	9 41 19	4	4.94 (3.51)	4.58 (0.27)	0.046
**B) Functional connectivity analysis (PPI)—Intermediate dissonance > consonance**					
Seed region (6mm radius sphere) located around highest peak for each subject within the right Heschl’s gyrus					
Temporal Sup. L	-51–16 4	8	4.72 (3.42)	3.99 (0.47)	0.043
Temporal Sup. R	60–7 1	23	4.08 (3.12)	3.38 (0.24)	0.027

**A)** Results (FWE-corrected *P* < 0.05 for cluster-level inference) of group General Linear Model for the contrasts: consonance > baseline, intermediate dissonance > baseline and strong dissonance > consonance, intermediate dissonance > consonance, strong dissonance > intermediate dissonance. **B)** Results of group psychophysiological interaction analysis (PPI) for the contrast: intermediate dissonance > consonance, with seed voxels located in the right Heschl’s gyrus (rHG). The regions described showed stronger positive functional connectivity with the rHG. Abbreviations: L: left, R: right.

**Fig 3:**
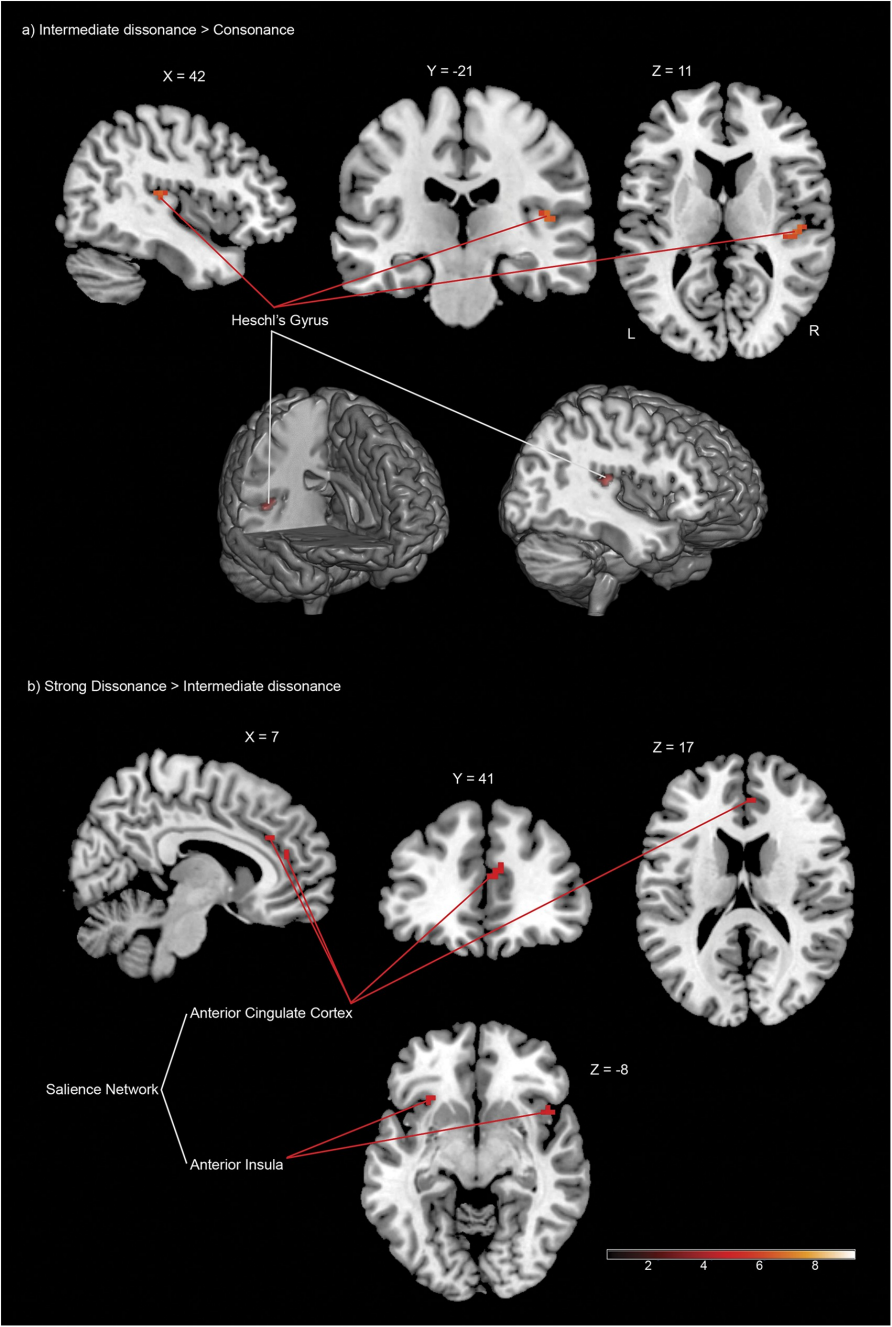
FMRI results (FWE-corrected P < 0.05 for cluster-level inference) [High resolution image]. Coloured areas (red) reflect: (a) Statistical parametric maps (SPM) showing voxels in the right Heschl’s gyrus in which the response was higher during the evaluation of intermediate dissonant sounds compared to consonant sounds, superimposed onto a standard brain in stereotactic MNI space (from left to right: sagittal, coronal and axial views; 3D render view below). (b) Statistical parametric maps showing voxels in right anterior cingulate cortex and bilateral anterior insula in which the response was higher during the evaluation of strong dissonant sounds compared to intermediate dissonant sounds.

The right anterior cingulate cortex (ACC) and the bilateral anterior insula (AI) showed increased activation whilst participants were evaluating the strong dissonant sounds compared to the intermediate dissonant sounds (see Table 4A and Fig 3B). Evidence indicates that strong dissonances could demand greater information integration due to their intrinsic complexity and negatively valenced appraisal [68,69,96], which segregates them as a motivationally significant stimuli. In agreement, previous studies suggest that the ACC and bilateral AI conform a salience network [97] that functions to identify salient and behaviourally relevant environmental stimuli for additional processing [98]. In the contrast between the strong dissonance and the consonance conditions, signal changes were observed within a cluster comprising the right angular gyrus (rAG) and the right inferior parietal cortex (see Table 4A), which could be interpreted as a modulation of stimulus-driven control of attention directed by strong dissonances, which elicited the most negatively valenced inferences and therefore signaled a behaviorally relevant event (the rAG is part of the “Ventral attention network” [26,99]). No significant signal changes were found when contrasting the consonance condition against intermediate dissonances.

Signal changes in the right Heschl’s gyrus were observed whilst participants were evaluating intermediate dissonant compared to consonant sounds (see Table 4A, Fig 3A). Considering that intermediate dissonances yielded averaged valence ratings between the strong dissonant and the consonant categories, which were supported by polynomial contrasts in both behavioral settings; and the fact that the intermediate dissonance category evinced the longest reaction times; we argue that the right Heschl’s gyrus response could indicate a perception bias towards sensory evidence, in an attempt to improve the quality of the stimulus representation in order to respond to the valence inference task under higher levels of predictive uncertainty [22,23].

**Fig 4:**
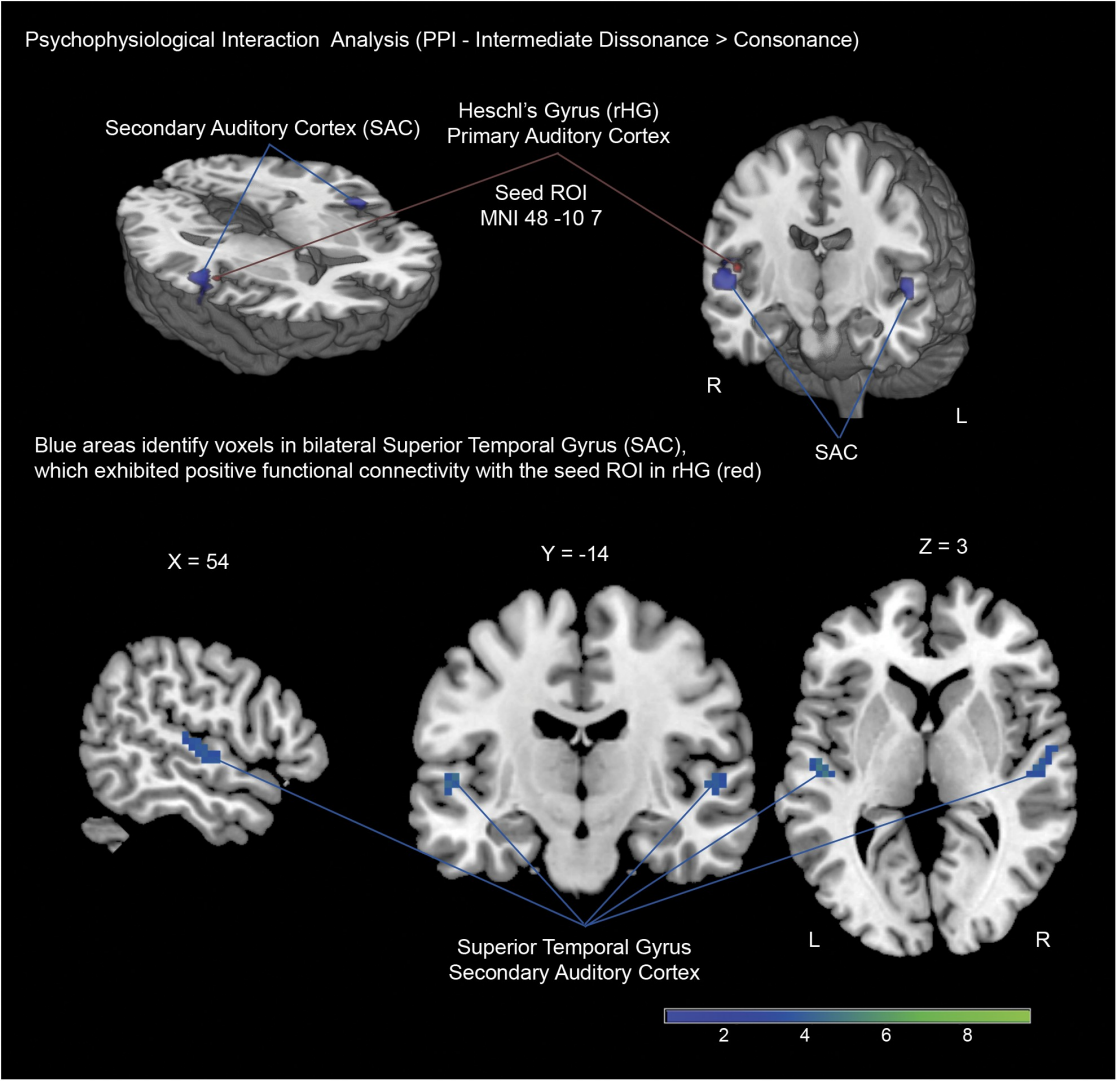
Psychophysiological Interaction Analysis: Blue color show voxels in bilateral Superior Temporal Gyrus (Secondary Auditory Cortex), which exhibited stronger functional connectivity with seed voxels (6mm sphere) located in the right Heschl’s gyrus (red) during the evaluation of intermediate dissonant compared with consonant sounds.

Functional connectivity (PPI) analysis was performed for the contrast between the intermediate dissonance condition and the consonance condition, with a seed region defined as a sphere with a 6mm radius around MNI coordinates -48–10 7 (group cluster peak activation in the right Heschl’s gyrus for the contrast intermediate dissonance > consonance, which was used as point of reference to identify individual subject activation peaks; see methods). A cluster comprising bilateral superior temporal gyrus exhibited stronger positive functional connectivity with the right Heschl’s gyrus, indicating a modulatory effect of intermediate dissonances in the interaction between the right Heschl’s gyrus and the secondary auditory cortex (bilaterally) (Table 4B, Fig 4). These results are consistent with cytoarchitectural findings reporting interaction between primary and secondary auditory cortices mediated by interhemispheric auditory pathways [100,101], and converge with evidence supporting an affective-attentional role of the auditory cortex when participants perform tasks that entail voluntary attention to auditory stimuli [102,103].

## Discussion

In the present experiment we examined the cognitive and neural mechanisms that underlie uncertainty with respect to emotional valence attribution to sound information, by investigating the effects of consonance/dissonance manipulation on participants’ recognition of affective cues in musical intervals. Consistent with previous studies [56,59,69,96], behavioural results indicated that participants rated the sounds that consist of more consonant intervals as having more positive valence, compared to sounds integrated by more dissonant intervals. Valence ratings for consonant intervals were significantly more positive than ratings for strong dissonant intervals in both behavioral settings (i.e. laboratory experiments and post-scan questionnaire). Intermediate dissonances yielded values between the strong dissonant and the consonant conditions, but which could not be clearly discriminated from either of these contrasting conditions. Polynomial contrasts further revealed a significant linear trend, showing that participants gave more extreme valence ratings to stimuli with more extreme consonant (or dissonant) interval content, whilst intermediate dissonances were rendered as the most ambiguous category in all behavioural experiments.

The results from the separate behavioral experiment conducted with a population similar to the fMRI participants in Buenos Aires (Argentina) upheld the results yielded by the experiment conducted in Cambridge (UK). These findings support previous empirical studies which have shown that, although the emotional appraisal of *tonal* dissonance seems to be strongly influenced by culture, as demonstrated by studies that have documented its variations across different cultures and its historical transformation through distinct Western culture periods [104]; judgments of *sensory* dissonance (the type of dissonance manipulated in the present paradigm) appear to be culturally invariant and largely independent of musical training [105]. On this regard, previous empirical findings have indicated that sensory dissonance could involve basic processing stages at a subcortical level (e.g. for the role of the inferior colliculus in the encoding of sensory dissonance see [54,56,63]) and have suggested that sensory dissonance could be less dependent on cultural learning ([58–62,64,65,70,75,106–109], although see also [110,111]).

Participant’s evaluation of consonances conveyed the fastest reaction times, and significantly faster than intermediate dissonances, which elicited the slowest reaction times. Only consonant intervals were given ratings that were significantly different from a neutral value, rendering consonances as the most salient condition with regards to valence rating. These findings are in agreement with the theoretical proposition argued by Schellenberg and collaborators concerning perceptual processing advantages for intervals with simple frequency ratios [68,75], which have been consistently found easier to encode, manage and recognise as a unit [68,75,112,113]. The neuroimaging experiment showed a general reduction of neural activity during valence judgments for consonances compared to intermediate and strong dissonances. These findings could be explained by such processing advantages of consonant sounds. The inherent complexity of strong dissonances may modulate brain systems involved in attention reorienting [99,114] and the detection of behaviourally relevant, salient and unexpected events [90,99]. In the present study this was supported by the engagement of bilateral AI and the ACC (“salience network” [98]) for the contrast comparing strong and intermediate dissonances (Table 4A and Fig 3B) and the involvement of the right inferior parietal cortex for the contrast between the strong dissonance condition and the consonance condition (“Ventral attention network” [99]) (Table 4A).

We consider that the longer reaction times and uncertain valence response for the intermediate dissonances could relate to the characteristic ambiguity and low salience of this sound category. The intermediate dissonance condition was constructed based on sequentially triggered minor thirds, a sonority which has been commonly applied to connote affective states of suspense and ambivalence in music [7,8]. From a music theory perspective, both the strong dissonance condition and the intermediate dissonance condition convey symmetrical forms [115,116]. The intermediate dissonance category, however, builds the content of a diminished seventh chord, whilst the strong dissonance condition assembles an octatonic scale (i.e. alternating intervals of whole and half step). When comparing both conditions, it should be noticed that the notes from the combination of exactly two diminished seventh chords (i.e. one additional rotation in the circle of fifths) would be required to obtain a the complete octatonic collection represented by the strong dissonance condition. Ambiguity appears as an essential characteristic of the diminished seventh chord. Because of its unpredictability, Arnold Schönberg has referred to it as a ‘vagrant’ chord [117]. Composers became aware of the expressive potential of this sonority, which was already employed in the Baroque era and frequently applied in the early years of the 19th century. Numerous examples of its use to represent unpredictable affective states are to be found in the operatic repertoire. The ambiguity of this intervallic content has been further underpinned in quantitative frameworks. Previous evidence indicates that the tonalness level of a sonority (i.e. degree to which a sonority evokes the perception of a clear tonal center) could represent a quantifiable predictor of emotional valence associations [72]. According to Temperley’s Bayesian key-finding model [76] the tonalness value for the intermediate dissonance category situates this sound condition in-between the other two extreme conditions (see Table 1), supporting its intermediate nature with regards to valence attribution.

We propose that a state of sensory attentiveness [22,23] might account for the significantly longer reaction times observed for evaluation of intermediate dissonances (compared to the consonant condition). It has been argued that stimuli with greater predictive uncertainty may suppress the use of top-down expectation driven information, because their predictive relationships with the environment are unknown [11]. The signal enhancement theory proposes that attention can improve the quality of the stimulus representation by increasing the gain of sensory-induced signals [4–6,118,119]. The fMRI experiment revealed signal changes in the right Heschl’s gyrus (primary auditory cortex) whilst participants were evaluating the intermediate dissonant condition, compared to the consonant condition (Fig 3A). We consider this finding to suggest a perception bias towards sensory evidence (i.e. turning up the ‘intensity’ of sensory channels) aimed at reducing the level of uncertainty for valence inferences within this category. The observation of a right-lateralized engagement is consistent with previous empirical evidence for preferential processing of pitch patterns and complex spectral information in the right primary auditory cortex [120–122]. The right Heschl’s gyrus is considered to have a selective role in the allocation of a spectral order to pitch information, which would allow to represent the amount of vibration at each individual frequency that is present in complex sounds [85,89,122–124]. In the context of our study, this functional specialization of the right Heschl’s gyrus could have been triggered to assist task performance during the evaluation of intermediate dissonances, prompting spectral processing mechanisms to acquire a more detailed acoustical analysis of the stimuli to respond to the valence inference task. Analogous cases of signal enhancement have been previously found in studies of covert attention within the visual domain, which have shown that attentional mechanisms can improve performance through amplification of the stimulus signal on the perceptual visual template, by multiplicatively increasing the gain of the neuronal response [5,6,118,119,125–130].

Signal changes in primary auditory cortices, including the right Heschl’s gyrus, were not observed for the contrast between the strong dissonance and the consonance categories, supporting the notion that the right Heschl’s gyrus activation was the strongest for the intermediate dissonance condition. However, signal changes were not found in this specific area for the comparison between intermediate and strong dissonances. It could be consequently argued that the right Heschl’s gyrus response might have been linked to the encoding of higher levels of dissonance in general, and not selectively to the processing of intermediate dissonances. However, the negative finding when contrasting the strong dissonance condition against the consonance condition would not be consistent with this alternate interpretation, since a greater effect size would be expected for strong dissonances compared to consonances if signal changes in this area would be generally modulated by increasing levels dissonance. We consider that the no significant difference in activation between intermediate and strong dissonances could have resulted from the common low emotional salience of both categories (their valence ratings were not significantly different from a neutral valence value). As evidenced in previous studies, dissonance (tonal or sensory aspects) generally renders emotional judgments to gravitate around a neutral value when compared with consonances, which normally convey a clear positively valenced appraisal [71,72]. It is important to note that, however, as shown in the present experiment, only the valence ratings for strong (and not intermediate) dissonances were significantly different from the ratings given to consonances. Interestingly, the reverse contrast (strong > intermediate dissonance) triggered a response of the salience network (Fig 3B) [98], showing that even within generally non-salient stimuli neural processes could still be initiated, possibly aimed at distinguishing and identifying the sound cues that elicited the most negatively valenced inferences, which could signal a behaviourally relevant event that requires to be marked and segregated for additional processing [90]. Taking together behavioural and neuroscientific findings, we argue that a coupling of these two attributes, the valence ambiguity and the low emotional salience associated with the intermediate dissonance category, may have elicited the response of the right Heschl’s gyrus during task performance.

To further examine possible modulatory influences of attentional mechanisms during participants’ evaluation of intermediate dissonances, functional connectivity (PPI) analysis was performed with a seed region defined around the highest peak of activation for each subject within the right Heschl’s gyrus (see methods). The results did not reveal a significant coupling (statistical dependence among remote neurophysiological events) between the right Heschl’s gyrus and any occipito-parietal cortices known to be involved in attention regulation [26,99]. However, the bilateral secondary auditory cortex evidenced a marked coactivation with the right Heschl’s gyrus when participants were evaluating intermediate dissonances compared to the consonance condition (Fig 4). These results are consistent with cytoarchitectural findings reporting interaction between primary and secondary auditory cortices mediated by interhemispheric auditory pathways running through the posterior third of the corpus callosum [100,101] and converge with evidence by Koelsch and collaborators [103], who have proposed that the auditory cortex may function as a central hub of an affective-attentional network. Following previous evidence for the role of the auditory cortex in selective attention, which has shown that participants’ voluntary attention to auditory stimuli may lead to a stronger primary and secondary auditory cortex activation [102,103], it is likely that our PPI results could be in part due to participants’ need to attain a more detailed acoustical analysis of intermediate dissonant sounds during task performance. The observed functional connectivity therefore highlights the role of primary-secondary auditory cortices’ interaction in the emotional processing of sound information, in particular during valence inferences for ambiguous and low salient auditory stimuli.

On balance, our findings show that the systematic manipulation of musical structural features can be applied to characterize the neurocognitive systems that underlie valence inference processes for low-salient and ambiguous musical intervals. A precise delineation of the mechanisms involved may have important implications for clinical neuroscience. In the context of mental state disorders, it has been shown that different psychopathologies react distinctively when faced with situations that entail uncertain outcomes [131,132]. For example, the attribution of a negative compared to a positive meaning to an ambiguous stimuli [132–134] is an important marker of negative mood and has been found to contribute to the development and maintenance of clinical depression and anxiety disorders [135,136]. Novel paradigms that manipulate uncertainty through non-verbal sound-based emotion recognition tasks could potentially be designed to measure biased information processing in individuals with specific language impairments, and further applied to clinical settings for psycho-diagnostic purposes.

### Future directions

Most of the previous empirical literature that has examined the neural basis of music-evoked emotions through the systematic control of musical dissonance has mainly focused on affective states elicited by extreme and contrasting levels of consonance/dissonance [69,96,137], and had rarely assessed subtle distinctions between emotions evoked by dissonances themselves. Our results showed that, although no differences were observed at a behavioral level (valence ratings) between strong and intermediate dissonances, the neuroscientific findings still revealed a modulation of attentional mechanisms directed by strong dissonances (i.e. response of the ventral attention and salience networks: [99]). Further research is therefore envisioned that might examine the musical dissonance valence percept as a behaviorally relevant event [26,98] in order to attain a finer characterization of brain activity in response to its emotional processing.

## Conclusions

Several studies have been conducted to investigate inferences made under uncertainty. However, no studies have yet examined the brain mechanisms that underlie uncertainty in the context of music-evoked emotions, and specifically, during the valence inferences to sound cues. Previous neuroimaging studies have shown enhanced activity in primary visual and auditory cortices under higher levels of predictive uncertainty. The present study was aimed at investigating the cognitive and neural mechanisms underlying uncertainty during the recognition of affective indices in musical intervals. We employed a sound-based emotion recognition paradigm in which participants categorized stimuli of three distinct levels of consonance/dissonance in terms of positive or negative valence. Behavioral results showed that, compared to consonances (perfect fourths, fifths and octaves) and strong dissonances (minor/major seconds and tritones), the intermediate dissonance category (minor thirds) elicited an ambiguous (i.e. uncertain valence) and low-salient response. Following the findings of the neuroscientific (fMRI) experiment, which showed an increased weight on perceptual sensory evidence (signal changes in the right Heschl’s gyrus) and a marked functional coupling with bilateral secondary auditory cortices whilst participants were evaluating intermediate dissonances compared to the consonant condition, we proposed that a state of sensory attentiveness could account for the significantly longer reaction times observed for the evaluation of this category. We argued that the inherent ambiguity and low emotional salience of the intermediate dissonance condition may have induced a heightened weight on evidence coming from sensory channels, in an attempt to obtain more detailed pitch pattern information (right Heschl’s gyrus functional specialization) in order to resolve the valence inference task.

Altogether, whilst consistent with previous studies showing enhanced sensory precision during perceptual uncertainty, our findings further extend this evidence to the evaluation of affective valence for cues signaled by musical intervals. We showed that an increased gain of sensory-induced signals might be initiated when subjects respond to low-salient stimuli with higher levels of predictive uncertainty during emotion recognition processes within the sound domain.

## Supporting information

S1 Table fMRI results (ANOVA). (DOCX)
