# Supplementary material for: Sensory cortical response to uncertainty and low salience during recognition of affective cues in musical intervals
Source: PLoS One. 2017 Apr 19;12(4):e0175991. doi: 10.1371/journal.pone.0175991 (PMC5396975; doi:10.1371/journal.pone.0175991)
Supplement: S1 Table — (DOCX) [file pone.0175991.s001.docx]

Supporting Information

**S1 Table. fMRI Results (ANOVA).**

| Region | Peak MNI | Voxels | Mean t (std.) | *p*-value (FWE) |
| --- | --- | --- | --- | --- |
| **ANOVA**  *(Main effect)* |  |  |  |  |
| **Cluster 1** |  |  |  |  |
| Insula L | -27 23 -8 | 262 | 20.52 (5.83) | < 0.001 |
| **Cluster 2** |  |  |  |  |
| Insula R | 48 14 -8 | 213 | 20.73 (5.45) | < 0.001 |
| Heschl R | 48 -19 10 | 67 | 53.28 (25.76) | < 0.001 |
| **Cluster 3** |  |  |  |  |
| Angular R | 36 -58 43 | 48 | 20.42 (5.35) | < 0.001 |
| Parietal inferior R | 36 -55 40 | 5 | 18.23 (3.31) | < 0.001 |
| **Cluster 4** |  |  |  |  |
| Anterior Cingulate R | 9 41 19 | 104 | 18.43 (3.19) | < 0.001 |

Results (FWE-corrected *P* < 0.05 for cluster-level inference). One-way ANOVA testing for overall main effects of the experimental manipulation (Conditions: strong dissonance, intermediate dissonance and consonance).

**References**

1. Bach DR, Dolan RJ. Knowing how much you don’t know: a neural organization of uncertainty estimates. Nat Rev Neurosci. 2012;13: 572–586. doi:10.1038/nrn3289

2. Dayan P, Yu AJ. Uncertainty and learning. IETE J Res. 2003;49: 171–181.

3. Pearce JM, Hall G. A model for Pavlovian learning: variations in the effectiveness of conditioned but not of unconditioned stimuli. Psychol Rev. 1980;87: 532.

4. Carrasco M. Spatial covert attention: Perceptual modulation. Oxf Handb Atten. 2014; 183–230.

5. Carrasco M, Williams PE, Yeshurun Y. Covert attention increases spatial resolution with or without masks: support for signal enhancement. J Vis. 2002;2: 467–479. doi:10.1167/2.6.4

6. Ling S, Carrasco M. When sustained attention impairs perception. Nat Neurosci. 2006;9: 1243–1245. doi:10.1038/nn1761

7. Huron D. Sweet Anticipation: Music and the Psychology of Expectation. Cambridge, Mass.: A Bradford Book; 2008.

8. Meyer L. Emotion and Meaning in Music [Internet]. 1956. Available: http://www.press.uchicago.edu/ucp/books/book/chicago/E/bo3643659.html

9. Battaglia PW, Jacobs RA, Aslin RN. Bayesian integration of visual and auditory signals for spatial localization. J Opt Soc Am A Opt Image Sci Vis. 2003;20: 1391–1397.

10. Clark JJ, Yuille AL. Data Fusion for Sensory Information Processing Systems [Internet]. Boston, MA: Springer US; 1990. Available: http://link.springer.com/10.1007/978-1-4757-2076-1

11. Dayan P, Kakade S, Montague PR. Learning and selective attention. Nat Neurosci. 2000;3 Suppl: 1218–1223. doi:10.1038/81504

12. Ernst MO, Banks MS. Humans integrate visual and haptic information in a statistically optimal fashion. Nature. 2002;415: 429–433. doi:10.1038/415429a

13. Knill DC, Richards W. Perception as Bayesian Inference [Internet]. 1996. Available: http://www.cambridge.org/us/academic/subjects/computer-science/computer-graphics-image-processing-and-robotics/perception-bayesian-inference

14. Yu AJ, Dayan P. Uncertainty, Neuromodulation, and Attention. Neuron. 2005;46: 681–692. doi:10.1016/j.neuron.2005.04.026

15. Friston KJ. The free-energy principle: a rough guide to the brain? Trends Cogn Sci. 2009;13: 293–301. doi:10.1016/j.tics.2009.04.005

16. Rao RPN, Ballard DH. Predictive coding in the visual cortex: a functional interpretation of some extra-classical receptive-field effects. Nat Neurosci. 1999;2: 79–87. doi:10.1038/4580

17. Dumoulin SO, Hess RF. Cortical specialization for concentric shape processing. Vision Res. 2007;47: 1608–1613. doi:10.1016/j.visres.2007.01.031

18. Fang F, Kersten D, Murray SO. Perceptual grouping and inverse fMRI activity patterns in human visual cortex. J Vis. 2008;8: 2. doi:10.1167/8.7.2

19. Murray SO, Kersten D, Olshausen BA, Schrater P, Woods DL. Shape perception reduces activity in human primary visual cortex. Proc Natl Acad Sci. 2002;99: 15164–15169. doi:10.1073/pnas.192579399

20. Feldman H, Friston KJ. Attention, uncertainty, and free-energy. Front Hum Neurosci. 2010;4: 215. doi:10.3389/fnhum.2010.00215

21. Kok P, Rahnev D, Jehee JFM, Lau HC, de Lange FP. Attention Reverses the Effect of Prediction in Silencing Sensory Signals. Cereb Cortex. 2012;22: 2197–2206. doi:10.1093/cercor/bhr310

22. Lawson RP, Rees G, Friston KJ. An aberrant precision account of autism. Front Hum Neurosci. 2014;8. doi:10.3389/fnhum.2014.00302

23. Boynton GM. A framework for describing the effects of attention on visual responses. Vision Res. 2009;49: 1129–1143. doi:10.1016/j.visres.2008.11.001

24. Brefczynski JA, DeYoe EA. A physiological correlate of the’spotlight’of visual attention. Nat Neurosci. 1999;2: 370–374.

25. Corbetta M, Shulman GL. Control of goal-directed and stimulus-driven attention in the brain. Nat Rev Neurosci. 2002;3: 215–229. doi:10.1038/nrn755

26. Corbetta M, Miezin FM, Dobmeyer S, Shulman GL, Petersen SE. Attentional modulation of neural processing of shape, color, and velocity in humans. Science. 1990;248: 1556–1559.

27. Gandhi SP, Heeger DJ, Boynton GM. Spatial attention affects brain activity in human primary visual cortex. Proc Natl Acad Sci. 1999;96: 3314–3319. doi:10.1073/pnas.96.6.3314

28. Martínez A, Anllo-Vento L, Sereno MI, Frank LR, Buxton RB, Dubowitz DJ, et al. Involvement of striate and extrastriate visual cortical areas in spatial attention. Nat Neurosci. 1999;2: 364–369. doi:10.1038/7274

29. Posner MI. Orienting of attention. Q J Exp Psychol. 1980;32: 3–25.

30. Reynolds JH, Heeger DJ. The Normalization Model of Attention. Neuron. 2009;61: 168–185. doi:10.1016/j.neuron.2009.01.002

31. Somers DC, Dale AM, Seiffert AE, Tootell RBH. Functional MRI reveals spatially specific attentional modulation in human primary visual cortex. Proc Natl Acad Sci. 1999;96: 1663–1668. doi:10.1073/pnas.96.4.1663

32. Alho K, Medvedev SV, Pakhomov SV, Roudas MS, Tervaniemi M, Reinikainen K, et al. Selective tuning of the left and right auditory cortices during spatially directed attention. Cogn Brain Res. 1999;7: 335–341. doi:10.1016/S0926-6410(98)00036-6

33. Alho K, Woods DL, Algazi A, Näätänen R. Intermodal selective attention. II. Effects of attentional load on processing of auditory and visual stimuli in central space. Electroencephalogr Clin Neurophysiol. 1992;82: 356–368.

34. Tzourio N, El Massioui F, Crivello F, Joliot M, Renault B, Mazoyer B. Functional Anatomy of Human Auditory Attention Studied with PET. NeuroImage. 1997;5: 63–77. doi:10.1006/nimg.1996.0252

35. Woodruff PW, Benson RR, Bandettini PA, Kwong KK, Howard RJ, Talavage T, et al. Modulation of auditory and visual cortex by selective attention is modality-dependent. Neuroreport. 1996;7: 1909–1913.

36. Woods DL, Alho K, Algazi A. Intermodal selective attention. I. Effects on event-related potentials to lateralized auditory and visual stimuli. Electroencephalogr Clin Neurophysiol. 1992;82: 341–355.

37. Van Overwalle F, Van den Eede S, Baetens K, Vandekerckhove M. Trait inferences in goal-directed behavior: ERP timing and localization under spontaneous and intentional processing. Soc Cogn Affect Neurosci. 2009;4: 177–190. doi:10.1093/scan/nsp003

38. Van Overwalle F. Social cognition and the brain: A meta-analysis. Hum Brain Mapp. 2009;30: 829–858. doi:10.1002/hbm.20547

39. Abell F, Happe F, Frith U. Do triangles play tricks? Attribution of mental states to animated shapes in normal and abnormal development. Cogn Dev. 2000;15: 1–16.

40. Castelli F, Frith C, Happé F, Frith U. Autism, Asperger syndrome and brain mechanisms for the attribution of mental states to animated shapes. Brain. 2002;125: 1839–1849. doi:10.1093/brain/awf189

41. Martin A, Weisberg J. Neural foundations for understanding social and mechanical concepts. Cogn Neuropsychol. 2003;20: 575–587. doi:10.1080/02643290342000005

42. Saxe, Wexler A. Making sense of another mind: The role of the right temporo-parietal junction. Neuropsychologia. 2005;43: 1391–1399. doi:10.1016/j.neuropsychologia.2005.02.013

43. Schultz J, Imamizu H, Kawato M, Frith CD. Activation of the human superior temporal gyrus during observation of goal attribution by intentional objects. J Cogn Neurosci. 2004;16: 1695–1705. doi:10.1162/0898929042947874

44. Gallagher HL, Happé F, Brunswick N, Fletcher PC, Frith U, Frith CD. Reading the mind in cartoons and stories: an fMRI study of “theory of mind”in verbal and nonverbal tasks. Neuropsychologia. 2000;38: 11–21.

45. Völlm BA, Taylor ANW, Richardson P, Corcoran R, Stirling J, McKie S, et al. Neuronal correlates of theory of mind and empathy: A functional magnetic resonance imaging study in a nonverbal task. NeuroImage. 2006;29: 90–98. doi:10.1016/j.neuroimage.2005.07.022

46. Helmholtz H von, Ellis AJ. On the sensations of tone as a physiological basis for the theory of music [Internet]. London, New York : Longmans, Green, and Co.; 1895. Available: http://archive.org/details/onsensationsofto00helmrich

47. Kameoka A, Kuriyagawa M. Consonance theory part II: consonance of complex tones and its calculation method. J Acoust Soc Am. 1969;45: 1460–1469.

48. Plomp R, Levelt WJM. Tonal consonance and critical bandwidth. J Acoust Soc Am. 1965;38: 548–560.

49. Terhardt E. Psychoacoustic evaluation of musical sounds. Percept Psychophys. 1978;23: 483–492. doi:10.3758/BF03199523

50. Terhardt E. The Concept of Musical Consonance: A Link between Music and Psychoacoustics. Music Percept Interdiscip J. 1984;1: 276–295. doi:10.2307/40285261

51. Zwicker E, Flottorp G, Stevens SS. Critical Band Width in Loudness Summation. J Acoust Soc Am. 1957;29: 548–557. doi:10.1121/1.1908963

52. Fletcher H. Auditory Patterns. Rev Mod Phys. 1940;12: 47–65. doi:10.1103/RevModPhys.12.47

53. Bidelman GM, Krishnan A. Neural Correlates of Consonance, Dissonance, and the Hierarchy of Musical Pitch in the Human Brainstem. J Neurosci. 2009;29: 13165–13171. doi:10.1523/JNEUROSCI.3900-09.2009

54. Cousineau M, McDermott JH, Peretz I. The basis of musical consonance as revealed by congenital amusia. Proc Natl Acad Sci. 2012;109: 19858–19863. doi:10.1073/pnas.1207989109

55. Fritz TH, Renders W, Müller K, Schmude P, Leman M, Turner R, et al. Anatomical differences in the human inferior colliculus relate to the perceived valence of musical consonance and dissonance. Eur J Neurosci. 2013; n/a-n/a. doi:10.1111/ejn.12305

56. McDermott JH, Lehr AJ, Oxenham AJ. Individual Differences Reveal the Basis of Consonance. Curr Biol. 2010;20: 1035–1041. doi:10.1016/j.cub.2010.04.019

57. Bidelman GM, Krishnan A. Brainstem correlates of behavioral and compositional preferences of musical harmony. Neuroreport. 2011;22: 212–216. doi:10.1097/WNR.0b013e328344a689

58. Foss AH, Altschuler EL, James KH. Neural correlates of the Pythagorean ratio rules. Neuroreport. 2007;18: 1521–1525.

59. Fujisawa TX, Cook ND. The perception of harmonic triads: an fMRI study. Brain Imaging Behav. 2011;5: 109–125. doi:10.1007/s11682-011-9116-5

60. Itoh K, Suwazono S, Nakada T. Cortical processing of musical consonance: an evoked potential study. Neuroreport. 2003;14: 2303–2306.

61. Itoh K, Suwazono S, Nakada T. Central auditory processing of noncontextual consonance in music: an evoked potential study. J Acoust Soc Am. 2010;128: 3781–3787. doi:10.1121/1.3500685

62. McKinney MF, Tramo MJ, Delgutte B. Neural correlates of musical dissonance in the inferior colliculus. Physiol Psychophys Bases Audit Funct Breebaart DJ Houtsma AJM Kohlrausch Prijs VF Schoonhoven R Eds. 2001; 83–89.

63. Minati L, Rosazza C, D’Incerti L, Pietrocini E, Valentini L, Scaioli V, et al. Functional MRI/event-related potential study of sensory consonance and dissonance in musicians and nonmusicians. Neuroreport. 2009;20: 87–92. doi:10.1097/WNR.0b013e32831af235

64. Peretz I, Blood AJ, Penhune V, Zatorre R. Cortical deafness to dissonance. Brain J Neurol. 2001;124: 928–940.

65. Soveri A, Tallus J, Laine M, Nyberg L, Bäckman L, Hugdahl K, et al. Modulation of Auditory Attention by Training. Exp Psychol. 2013;60: 44–52. doi:10.1027/1618-3169/a000172

66. Ayres T, Aeschbach S, Walker EL. Psychoacoustic and experiential determinants of tonal consonance. J Aud Res. 1980;20: 31–42.

67. Schellenberg, Trehub SE. Frequency ratios and the discrimination of pure tone sequences. Percept Psychophys. 1994;56: 472–478. doi:10.3758/BF03206738

68. Blood AJ, Zatorre RJ, Bermudez P, Evans AC. Emotional responses to pleasant and unpleasant music correlate with activity in paralimbic brain regions. Nat Neurosci. 1999;2: 382–387. doi:10.1038/7299

69. Zentner MR, Kagan J. Perception of music by infants. Nature. 1996;383: 29–29. doi:10.1038/383029a0

70. Bravo F. The Influence of Music on the Emotional Interpretation of Visual Contexts. In: Aramaki M, Barthet M, Kronland-Martinet R, Ystad S, editors. From Sounds to Music and Emotions. Springer Berlin Heidelberg; 2012. pp. 366–377. doi:10.1007/978-3-642-41248-6_20

71. Bravo F. Changing the Interval Content of Algorithmically Generated Music Changes the Emotional Interpretation of Visual Images. In: Aramaki M, Derrien O, Kronland-Martinet R, Ystad S, editors. Sound, Music, and Motion. Cham: Springer International Publishing; 2014. pp. 494–508. Available: http://link.springer.com/10.1007/978-3-319-12976-1_29

72. Piston W. Harmony: Fifth Edition. 5 edition. DeVoto M, editor. New York: W. W. Norton & Company; 1987.

73. Schönberg A. Harmonielehre. 7 edition. Place of publication not identified: Universal Edition; 1966.

74. Schellenberg EG, Trainor LJ. Sensory consonance and the perceptual similarity of complex-tone harmonic intervals: tests of adult and infant listeners. J Acoust Soc Am. 1996;100: 3321–3328.

75. Temperley D. Music and Probability. The MIT Press; 2010.

76. Hansen KA, Hillenbrand SF, Ungerleider LG. Effects of Prior Knowledge on Decisions Made Under Perceptual vs. Categorical Uncertainty. Front Neurosci. 2012;6. doi:10.3389/fnins.2012.00163

77. DeWitt LA, Crowder RG. Tonal fusion of consonant musical intervals: the oomph in Stumpf. Percept Psychophys. 1987;41: 73–84.

78. Vos J, Vianen BG van. The effect of fundamental frequency on the discriminability between pure and tempered fifths and major thirds. Percept Psychophys. 1984;37: 507–514. doi:10.3758/BF03204914

79. Parncutt R. Harmony: a psychoacoustical approach. Springer-Verlag; 1989.

80. Krumhansl CL. Cognitive Foundations of Musical Pitch. New York: Oxford University Press; 2001.

81. Moore BCJ. Frequency difference limens for short‐duration tones. J Acoust Soc Am. 1973;54: 610–619. doi:10.1121/1.1913640

82. Turnbull WW. Pitch discrimination as a function of tonal duration. J Exp Psychol. 1944;34: 302–316. doi:10.1037/h0063434

83. Friston KJ, Ashburner J, Frith CD, Poline J-B, Heather JD, Frackowiak RSJ. Spatial registration and normalization of images. Hum Brain Mapp. 1995;3: 165–189. doi:10.1002/hbm.460030303

84. Johnsrude IS, Penhune VB, Zatorre RJ. Functional specificity in the right human auditory cortex for perceiving pitch direction. Brain J Neurol. 2000;123 ( Pt 1): 155–163.

85. Liégeois-Chauvel C, Giraud K, Badier J-M, Marquis P, Chauvel P. Intracerebral Evoked Potentials in Pitch Perception Reveal a Functional Asymmetry of the Human Auditory Cortex. Ann N Y Acad Sci. 2001;930: 117–132. doi:10.1111/j.1749-6632.2001.tb05728.x

86. Patterson RD, Uppenkamp S, Johnsrude IS, Griffiths TD. The Processing of Temporal Pitch and Melody Information in Auditory Cortex. Neuron. 2002;36: 767–776. doi:10.1016/S0896-6273(02)01060-7

87. Warrier C, Wong P, Penhune V, Zatorre R, Parrish T, Abrams D, et al. Relating Structure to Function: Heschl’s Gyrus and Acoustic Processing. J Neurosci. 2009;29: 61–69. doi:10.1523/JNEUROSCI.3489-08.2009

88. Zatorre RJ. Pitch perception of complex tones and human temporal-lobe function. J Acoust Soc Am. 1988;84: 566–572.

89. Uddin LQ. Salience processing and insular cortical function and dysfunction. Nat Rev Neurosci. 2015;16: 55–61. doi:10.1038/nrn3857

90. Maldjian JA, Laurienti PJ, Kraft RA, Burdette JH. An automated method for neuroanatomic and cytoarchitectonic atlas-based interrogation of fMRI data sets. NeuroImage. 2003;19: 1233–1239.

91. Friston KJ, Buechel C, Fink GR, Morris J, Rolls E, Dolan RJ. Psychophysiological and modulatory interactions in neuroimaging. Neuroimage. 1997;6: 218–229.

92. Loftus GR, Masson ME. Using confidence intervals in within-subject designs. Psychon Bull Rev. 1994;1: 476–490.

93. Menon. Neural Correlates of Timbre Change in Harmonic Sounds. NeuroImage. 2002;17: 1742–1754. doi:10.1006/nimg.2002.1295

94. Ohnishi T, Matsuda H, Asada T, Aruga M, Hirakata M, Nishikawa M, et al. Functional Anatomy of Musical Perception in Musicians. Cereb Cortex. 2001;11: 754–760. doi:10.1093/cercor/11.8.754

95. Koelsch S, Fritz T, v. Cramon DY, Müller K, Friederici AD. Investigating emotion with music: An fMRI study. Hum Brain Mapp. 2006;27: 239–250. doi:10.1002/hbm.20180

96. Seeley WW, Menon V, Schatzberg AF, Keller J, Glover GH, Kenna H, et al. Dissociable intrinsic connectivity networks for salience processing and executive control. J Neurosci Off J Soc Neurosci. 2007;27: 2349–2356. doi:10.1523/JNEUROSCI.5587-06.2007

97. Menon V, Uddin LQ. Saliency, switching, attention and control: a network model of insula function. Brain Struct Funct. 2010;214: 655–667. doi:10.1007/s00429-010-0262-0

98. Corbetta M, Patel G, Shulman GL. The reorienting system of the human brain: from environment to theory of mind. Neuron. 2008;58: 306–324. doi:10.1016/j.neuron.2008.04.017

99. Aboitiz F, Scheibel AB, Fisher RS, Zaidel E. Fiber composition of the human corpus callosum. Brain Res. 1992;598: 143–153.

100. Bamiou D-E, Sisodiya S, Musiek FE, Luxon LM. The role of the interhemispheric pathway in hearing. Brain Res Rev. 2007;56: 170–182. doi:10.1016/j.brainresrev.2007.07.003

101. Jäncke L, Mirzazade S, Shah NJ. Attention modulates activity in the primary and the secondary auditory cortex: a functional magnetic resonance imaging study in human subjects. Neurosci Lett. 1999;266: 125–128.

102. Koelsch S, Skouras S, Fritz T, Herrera P, Bonhage C, Küssner MB, et al. The roles of superficial amygdala and auditory cortex in music-evoked fear and joy. NeuroImage. 2013;81: 49–60. doi:10.1016/j.neuroimage.2013.05.008

103. Burns EM. 7 - Intervals, Scales, and Tuning*. In: Deutsch D, editor. The Psychology of Music (Second Edition). San Diego: Academic Press; 1999. pp. 215–264. doi:10.1016/B978-012213564-4/50008-1

104. Butler JW, Daston PG. Musical consonance as musical preference: a cross-cultural study. J Gen Psychol. 1968;79: 129–142. doi:10.1080/00221309.1968.9710460

105. Fannin HA, Braud WG. Preference for Consonant over Dissonant Tones in the Albino Rat. Percept Mot Skills. 1971;32: 191–193. doi:10.2466/pms.1971.32.1.191

106. Izumi A. Japanese monkeys perceive sensory consonance of chords. J Acoust Soc Am. 2000;108: 3073–3078.

107. Sugimoto T, Kobayashi H, Nobuyoshi N, Kiriyama Y, Takeshita H, Nakamura T, et al. Preference for consonant music over dissonant music by an infant chimpanzee. Primates J Primatol. 2010;51: 7–12. doi:10.1007/s10329-009-0160-3

108. Chiandetti C, Vallortigara G. Chicks like consonant music. Psychol Sci. 2011;22: 1270–1273. doi:10.1177/0956797611418244

109. McDermott J, Hauser M. Are consonant intervals music to their ears? Spontaneous acoustic preferences in a nonhuman primate. Cognition. 2004;94: B11-21. doi:10.1016/j.cognition.2004.04.004

110. McDermott JH, Schultz AF, Undurraga EA, Godoy RA. Indifference to dissonance in native Amazonians reveals cultural variation in music perception. Nature. 2016; doi:10.1038/nature18635

111. Bharucha JJ, Pryor JH. [Disrupting the isochrony underlying rhythm: an asymmetry in discrimination]. Percept Psychophys. 1986;40: 137–141.

112. Francès R. La perception de la musique. Vrin; 1984.

113. Chambers CD, Payne JM, Stokes MG, Mattingley JB. Fast and slow parietal pathways mediate spatial attention. Nat Neurosci. 2004;7: 217–218. doi:10.1038/nn1203

114. Cohn R. Audacious Euphony: Chromatic Harmony and the Triad’s Second Nature. Oxford University Press, USA; 2012.

115. Tymoczko D. A Geometry of Music: Harmony and Counterpoint in the Extended Common Practice. Oxford University Press, USA; 2011.

116. Schoenberg A. Theory of Harmony. University of California Press; 1983.

117. Dosher BA, Lu Z-L. Noise exclusion in spatial attention. Psychol Sci. 2000;11: 139–146.

118. Lu Z-L, Dosher BA. External noise distinguishes mechanisms of attention. Vis Res. 1998;38: 1183–1198.

119. Petkov CI, Kang X, Alho K, Bertrand O, Yund EW, Woods DL. Attentional modulation of human auditory cortex. Nat Neurosci. 2004;7: 658–663. doi:10.1038/nn1256

120. Johnsrude IS, Penhune VB, Zatorre RJ. Functional specificity in the right human auditory cortex for perceiving pitch direction. Brain J Neurol. 2000;123 ( Pt 1): 155–163.

121. Zatorre RJ, Belin P, Penhune VB. Structure and function of auditory cortex: music and speech. Trends Cogn Sci. 2002;6: 37–46.

122. Liégeois-Chauvel C, Giraud K, Badier J-M, Marquis P, Chauvel P. Intracerebral Evoked Potentials in Pitch Perception Reveal a Functional Asymmetry of the Human Auditory Cortex. Ann N Y Acad Sci. 2001;930: 117–132. doi:10.1111/j.1749-6632.2001.tb05728.x

123. Patterson RD, Uppenkamp S, Johnsrude IS, Griffiths TD. The processing of temporal pitch and melody information in auditory cortex. Neuron. 2002;36: 767–776.

124. Cameron EL, Tai JC, Carrasco M. Covert attention affects the psychometric function of contrast sensitivity. Vision Res. 2002;42: 949–967.

125. Carrasco M, Penpeci-Talgar C, Eckstein M. Spatial covert attention increases contrast sensitivity across the CSF: support for signal enhancement. Vision Res. 2000;40: 1203–1215.

126. Bashinski HS, Bacharach VR. Enhancement of perceptual sensitivity as the result of selectively attending to spatial locations. Percept Psychophys. 1980;28: 241–248. doi:10.3758/BF03204380

127. Downing CJ. Expectancy and visual-spatial attention: effects on perceptual quality. J Exp Psychol Hum Percept Perform. 1988;14: 188–202.

128. Luck SJ, Hillyard SA, Mouloua M, Hawkins HL. Mechanisms of visual-spatial attention: resource allocation or uncertainty reduction? J Exp Psychol Hum Percept Perform. 1996;22: 725–737.

129. Morrone MC, Denti V, Spinelli D. Color and Luminance Contrasts Attract Independent Attention. Curr Biol. 2002;12: 1134–1137. doi:10.1016/S0960-9822(02)00921-1

130. Berna C, Lang TJ, Goodwin GM, Holmes EA. Developing a measure of interpretation bias for depressed mood: An ambiguous scenarios test. Personal Individ Differ. 2011;51: 349–354. doi:10.1016/j.paid.2011.04.005

131. Rude SS, Wenzlaff RM, Gibbs B, Vane J, Whitney T. Negative processing biases predict subsequent depressive symptoms. Cogn Emot. 2002;16: 423–440. doi:10.1080/02699930143000554

132. Butler G, Mathews A. Cognitive Processes in Anxiety. ResearchGate. 1983;5: 51–62. doi:10.1016/0146-6402(83)90015-2

133. Lawson C, MacLeod C, Hammond G. Interpretation revealed in the blink of an eye: Depressive bias in the resolution of ambiguity. J Abnorm Psychol. 2002;111: 321–328. doi:10.1037/0021-843X.111.2.321

134. Beck AT. Cognitive Therapy and the Emotional Disorders. London: Penguin Books, Limited; 1991.

135. Mathews A, MacLeod C. Cognitive Vulnerability to Emotional Disorders. Annu Rev Clin Psychol. 2005;1: 167–195. doi:10.1146/annurev.clinpsy.1.102803.143916

136. Gosselin N, Samson S, Adolphs R, Noulhiane M, Roy M, Hasboun D, et al. Emotional responses to unpleasant music correlates with damage to the parahippocampal cortex. Brain. 2006;129: 2585–2592. doi:10.1093/brain/awl240
